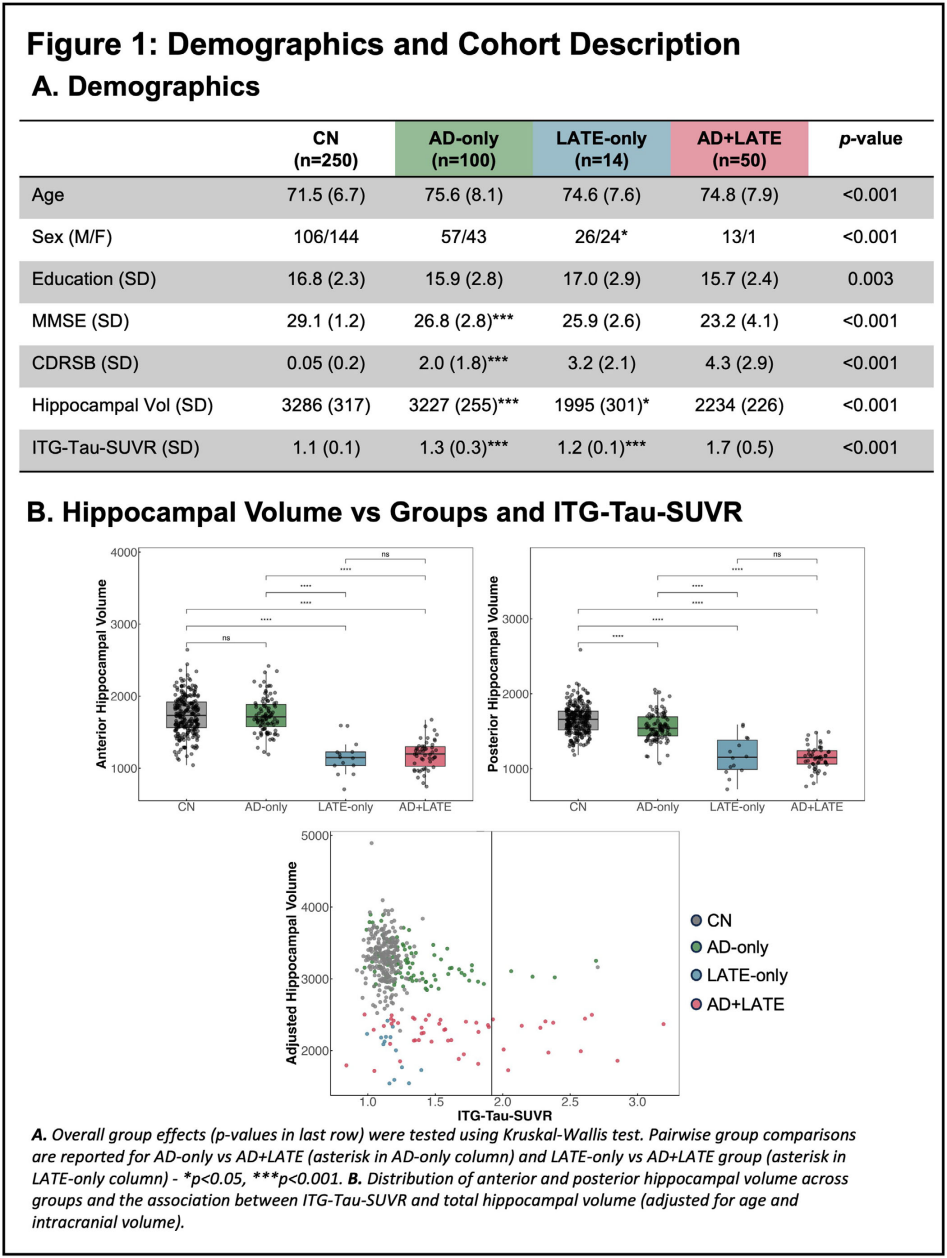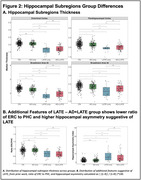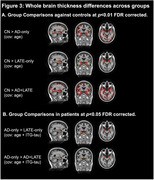# Percentile‐based hippocampal volume MRI biomarker for distinguishing concomitant LATE and AD from pure LATE and pure AD

**DOI:** 10.1002/alz.095151

**Published:** 2025-01-09

**Authors:** Nidhi S. Mundada, Xueying Lyu, Emily McGrew, Long Xie, Paul A. Yushkevich, Sandhitsu R. Das, David A Wolk

**Affiliations:** ^1^ University of Pennsylvania, Philadelphia, PA USA

## Abstract

**Background:**

Due to the lack of *in vivo* molecular biomarkers for Limbic‐predominant age‐related TDP‐43 encephalopathy (LATE), which commonly co‐occurs with Alzheimer’s disease (AD), it is challenging to identify patients with mixed AD/LATE pathology. Autopsy studies have suggested that AD patients with greater hippocampal atrophy are disproportionately enriched in having concomitant LATE. Here we define the lower quartile of hippocampal volume as a biomarker of possible LATE with AD.

**Method:**

We identified 164 cognitively impaired participants from ADNI who had structural MRI, amyloid‐PET, and tau‐PET within 365 days. Patients were grouped based on hippocampal volume quartiles (adjusted for age and intracranial volume) and amyloid‐PET‐status into *suspected* 1) AD‐only (volume>50th‐percentile, amyloid‐positive), n = 100, 2) LATE‐only (volume<25th‐percentile, amyloid‐negative), n = 14, 3) AD+LATE (volume<25th‐percentile, amyloid‐positive), n = 50. 250 cognitively normal, amyloid‐negative participants were also included. Patterns of atrophy were examined to assess whether the AD+LATE group had features suggestive of LATE beyond hippocampal atrophy.

**Result:**

Suspected‐AD+LATE group had lower MMSE, CDR, and hippocampal volume and higher ITG‐tau‐SUVR compared to the suspected‐AD‐only group (Figure‐1A). Suspected‐AD‐only group showed predominant posterior hippocampal atrophy whereas LATE showed a more severe anterior hippocampal atrophy (Figure‐1B/2B). When compared to the AD‐only group, suspected LATE‐only and AD+LATE groups showed significantly lower thickness in ERC, PHC, BA35, BA36 (Figure‐2A); there were no differences between LATE‐only and AD+LATE groups. Suspected‐AD+LATE group showed features suggestive of LATE in prior work (Figure‐2B). Whole brain analysis demonstrated that in comparison to controls the suspected‐AD‐only group showed lower thickness in typical AD regions (posterior hippocampus and temporoparietal regions), suspected‐LATE‐only group showed lower thickness in more anterior MTL regions including temporal pole consistent with LATE pathology, whereas suspected‐AD+LATE group showed lower thickness in both AD‐ and LATE‐like regions (Figure‐3A). Assessing neurodegeneration driven due to non‐tau factors by controlling for ITG‐tau‐SUVR revealed similar atrophy patterns for both suspected LATE‐only and AD+LATE groups compared to the AD‐only group. Both groups showed decreased thickness isolated to anterior hippocampus extending into temporal pole (Figure‐3B).

**Conclusion:**

Using a simple hippocampal volume percentile‐based metric may enrich in patients with concomitant LATE on the AD continuum and could potentially be examined in the context of clinical trials and anti‐amyloid therapies.